# Chemical Composition and Biological Activities of *Centranthus longiflorus* Stems Extracts Recovered Using *Ired-Irrad*^®^, an Innovative Infrared Technology, Compared to Water Bath and Ultrasound

**DOI:** 10.3390/life13061288

**Published:** 2023-05-30

**Authors:** Mariam Hammoud, Hiba N. Rajha, Ali Chokr, Carl Safi, Lambertus A. M. van den Broek, Gijs van Erven, Richard G. Maroun, Espérance Debs, Hassan Rammal, Nicolas Louka

**Affiliations:** 1 Centre d’Analyses et de Recherche, Unité de Recherche Technologies et Valorisation Agro-Alimentaire, Faculté des Sciences, Université Saint-Joseph de Beyrouth, CST-Mar Roukos-Dekwaneh, Riad El Solh, P.O. Box 1514, Beirut 1107 2050, Lebanon; mariam.hammoud2@net.usj.edu.lb (M.H.);; 2 Research Laboratory of Microbiology (RLM), Department of Life and Earth Sciences, Faculty of Sciences I, Lebanese University, Hadath Campus, P.O. Box 5, Beirut 1683, Lebanon; 3 Platform of Research and Analysis in Environmental Sciences (PRASE), Doctoral School of Sciences and Technology (DSST), Lebanese University, Hadath Campus, P.O. Box 6573/14, Beirut 1683, Lebanon; 4 Ecole Supérieure d’Ingénieurs de Beyrouth (ESIB), Université Saint-Joseph de Beyrouth, CST-Mar Roukos-Dekwaneh, Riad El Solh, P.O. Box 1514, Beirut 1107 2050, Lebanon; 5 Wageningen Food & Biobased Research, P.O. Box 17, 6700 AA Wageningen, The Netherlands; 6 Department of Biology, Faculty of Arts and Sciences, University of Balamand, P.O. Box 100, Tripoli 1300, Lebanon; esperance.debs@balamand.edu.lb

**Keywords:** *Centranthus longiflorus*, phenolic compounds, *Ired-Irrad* ^®^, ultrasound, antibacterial activity

## Abstract

Extraction of polyphenols from *Centranthus longiflorus* stems was conducted using ultrasound and infrared *Ired-Irrad*^®^ techniques, and compared to the conventional water bath method. Response surface methodology was used to analyse the effect of time, temperature, and ethanol percentage, as well as to optimize the three extraction methods. The highest phenolic content (81 mg GAE/g DM) and antioxidant activity (76% DPPH inhibition) were recorded with the *Ired-Irrad*^®^ extract obtained under the optimal conditions: 55 °C, 127 min, 48% (*v*/*v*) ethanol. Biological activities (antioxidant, antibacterial and antibiofilm) of the three extracts were assessed. All *C*. *longiflorus* stems extracts showed limited antibacterial effects regardless of the extraction method (MIC = 50 mg/mL), whereas *Ired-Irrad*^®^ extract exhibited the highest biofilm eradication and prevention capacities (93% against *Escherichia coli* and 97% against *Staphylococcus epidermidis*, respectively). This bioactivity is likely related to abundant caffeoylquinic acid and quercetin rutinoside, as identified by RP-UHPLC-PDA-MS analysis. The results obtained further promote the effectiveness of *Ired-Irrad*^®^ as a highly flexible and cost-efficient extraction technique.

## 1. Introduction

Many plant species are well known for their medicinal value and promising therapeutic potential. They are considered as a valuable source for the investigation of new bioactive compounds that can be used in food, pharmaceutical, and cosmetics industries. Due to the increasing demand for natural remedies nowadays, the interest in medicinal plants has grown globally [1]. Several studies have confirmed the biological [2] and pharmacological properties of plant-derived compounds, such as anti-inflammatory, antioxidant, antibacterial, and antiviral effects [3]. Such potent biological activities have been primarily related to the phytochemical profile, mainly phenolics, alkaloids, flavonoids, and many other secondary metabolites.

Extraction of bioactive compounds from plant matrices is a crucial step toward their recovery, since the selected method plays a key role in their subsequent separation and characterization. Water bath extraction is the most conventional method used. It is based on the diffusion of target molecules from plant materials into an aqueous or organic solvent phase. It is commonly used, mainly thanks to its ease of use and wide range of applicability [4]. Nonetheless, it is usually associated with the consumption of large volumes of solvents, extended extraction times, and the possible thermal degradation of the bioactive molecules [5]. Therefore, more modern assisted extraction techniques which reduce time and organic solvent consumption, and increase extraction efficiency, have been developed, e.g., ultrasound, pulsed-electric field, microwave, supercritical fluid, pressurized liquid, high-voltage electrical discharges, and many others.

Ultrasound permits cell disruption through cell membrane alteration, which facilitates the diffusion of solvent into the cells, thereby leading to a higher extraction yield [6]. This extraction technique is based on the use of an ultrasonic bath with ultrasound frequency, temperature, and extraction time being controlled. On the other hand, infrared irradiation is introduced as a green extraction technique used for maximizing the recovered fraction [7]. *Ired-Irrad*^®^ is a prototype provided with an emitter of infrared irradiation, that enhances the yield of extraction. This eco-friendly technique has been explored; it allowed the intensification of polyphenol recovery compared to conventional methods.

About 2600 wild plant species, including 92 endemic ones, can be found in Lebanon, among which, is the *Centranthus longiflorus* L. plant. The genus *Centranthus* belongs to the *Caprifoliaceae* family, recognized as the red valerian [8]. Several studies have confirmed the antioxidant and antibacterial activities of the *C. longiflorus* compounds extracted by conventional methods [9]. The extracts of *C. longiflorus* are known for their abundance in secondary metabolites, which have several medical applications such as analgesic activity due to the presence of alkaloids, antioxidant activity thanks to their phenolic compounds, and antitumor activity owing to their flavonoids [10]. Hence, the main objective of this study was to extract polyphenols from *C*. *longiflorus* stems using two emerging techniques, infrared and ultrasound, and the conventional water bath for comparison. Antioxidant, antibacterial, and antibiofilm activities of the different extracts were assessed.

## 2. Materials and Methods

### 2.1. Plant Material Preparation

*Centranthus longiflorus* was collected between June and July 2019 (900 m altitude) from Mount Lebanon (Arz el Bâroûk—33°40′60″ N–35°40′60″ E). The plant genus and species were confirmed using Lebanon’s flora guide [8]. Stems were well cleaned and dried at 35°C for 48 h in an airflow oven (UFE 700, Memmert GmbH, Schwabach, Germany). Dried stems were ground and stored in the dark at room temperature until further use.

### 2.2. Dry Matter Content

Dry matter (DM) was determined after totally drying fresh *C. longiflorus* stems for 24 h in a ventilated oven at 105 °C [11]. DM of raw material was 90%.

### 2.3. Chemicals, Bacterial Strains and Media

All chemicals and materials used were of analytical grade. Gallic acid, sodium carbonate (Na_2_CO_3_), Folin–Ciocalteu reagent, 2,2-diphenyl-1-picrylhydrazyl (DPPH), and Trolox, as well as all solvents used for extraction experiments were purchased from Sigma-Aldrich, Steinheim, Germany.

Four bacterial strains were adopted to determine the antibacterial activity of *C. longiflorus* stems extracts, two Gram-positive, *Staphylococcus aureus* (ATCC 49619) and *Staphylococcus epidermidis* RP62A (ATCC 35984), and two Gram-negative, *Escherichia coli* (ATCC 35218) and *Pseudomonas aeruginosa* (ATCC 27853). All bacterial strains were stored in glycerol at −80 °C before use. The media brain heart agar (BHA), Luria–Bertani broth, Mueller–Hinton broth (MHB), and tryptic soy broth (TSB) were purchased from HIMEDIA (Mumbai, India) and prepared according to the manufacturer’s instructions.

### 2.4. Experimental Protocol

#### 2.4.1. Extraction Parameters

The following procedure applies to the three extractions methods. Ten grams of dried *C. longiflorus* stems were mixed with ethanol-water solvent (100 mL) with varying percentages of ethanol (as indicated in Section 2.4.5). Preliminary experiments were conducted to determine the optimal particle size and solid to liquid ratio (as indicated in Section 3.1). Extracts were filtered through glass wool, and concentrated using a rotary evaporator (Heidolph, Schwabach, Germany) before being lyophilized (CHRIST, Alpha 1-4 LD plus, Osterode am Harz, Germany) and used to assess the antibacterial activity.

#### 2.4.2. Water Bath Extraction (WB)

In a glass flask, a certain mass of dried *C. longiflorus* stems was mixed with the solvent. The flask was placed in a water bath (Clifton, Bristol, UK), and heated during a provided time at the indicated temperature.

#### 2.4.3. Ultrasound-Assisted Extraction (US)

An ultrasonic bath (Wise Clean Digital Ultrasonic Cleaner, 290 W, Wertheim, Germany) with temperature control was used to treat the samples of dried *C. longiflorus* stems with the solvent. Different frequencies (ranging from 10 to 40 kHz) were tested to choose the optimal one.

#### 2.4.4. Infrared-Assisted Extraction (IR)

**Figure 1:**
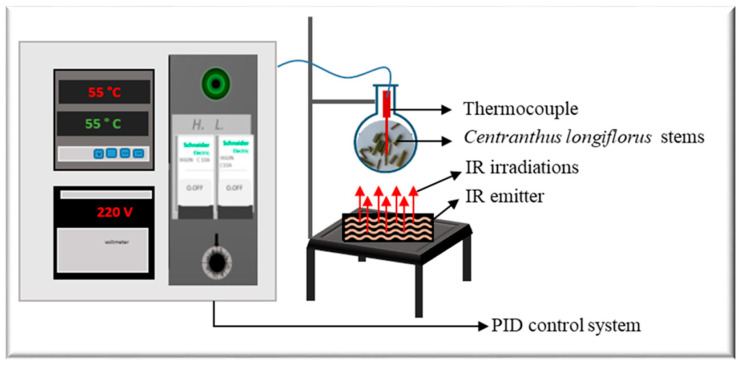
Infrared-assisted extraction setup.

The apparatus *Ired-Irrad*^®^ [12] was used for the infrared-assisted extraction (Patent 2017/11-11296L) of ground *C. longiflorus* stems (Figure 1). The prototype consists of a ceramic emitter (Rotfil, Pianezza, Italy), connected to a PID (proportional-integral-derivative) system for voltage and temperature control. Ground *C. longiflorus* stems were mixed with the solvent (ethanol-water) in a round bottom flask that was positioned at a 1 cm distance from the ceramic IR emitter.

#### 2.4.5. Experimental Design

The extraction process was optimized using Response Surface Methodology (RSM). Linear and quadratic effects of the three independent variables: extraction time “t”, extraction temperature “T”, and ethanol percentage “E”, were studied as well as their interactions. A central composite design (2^3^ + star), including 22 runs with eight repetitions at the central points, was drawn to assess the effect of the three studied parameters on TPC and DPPH inhibition percentage as response parameters. The three parameters varied between five levels coded −α, −1, 0, +1, +α: 36 min, 70 min, 120 min, 170 min and 204 min for extraction time, 21 °C, 35 °C, 55 °C, 75 °C and 88 °C for extraction temperature, and 16%, 30%, 50%, 70% and 83% for ethanol percentage. The same experimental design was applied on the three extraction techniques WB, US, and IR.

Experimental data were fitted to a second degree regression equation as follows in order to predict the response parameter Y:

Y = ε_0_ + ε_1_·t + ε_2_·T + ε_3_·E + ε_4_·t^2^ + ε_5_·T^2^ + ε_6_·E^2^ + ε_7_·t·T + ε_8_·t·E + ε_9_·T·E (1)

where the mean value of responses at the central point is ε_0_; the linear coefficients are ε_1_ to ε_3_, the quadratic coefficients are ε_4_ to ε_6_, the interaction coefficients are ε_7_ to ε_9_. The statistical analysis of the results and experimental design were done using STATGRAPHICS Centurion XVII-X64 (The Plains, VA, USA).

### 2.5. Quantification of Total Phenolic Content (TPC)

The Folin–Ciocalteu method as described by Singleton et al. [13] was applied. *C. longiflorus* stems extract (0.2 mL) was added to 1 mL of diluted Folin–Ciocalteu reagent (1/10 *v*/*v*) and 0.8 mL of Na_2_CO_3_ (7.5% *w*/*v*). The mixture was incubated at 60 °C for 10 min, and subsequently 10 min in a refrigerator. The absorbance was measured using a UV-Vis spectrophotometer (GENESYS 10 UV, Thermo Electron Corporation, Waltham, MA, USA) at 750 nm. Gallic acid was used for the calibration curve, and TPC was expressed in mg of Gallic Acid Equivalent per gram of DM (mg GAE/g DM).

### 2.6. Evaluation of the Antioxidant Activities

Four complementary assays were used to assess different aspects of antioxidant capacity of the extracts. Antioxidant activity of polyphenols in the samples, or their capacity to reduce stable free radicals was evaluated by both DPPH [14] and ABTS (2,2′-azinobis (3-ethylbenzothiazoline-6-sulphonic acid) assay kit) (Bioquochem, Asturias, Spain) [12]. In the DPPH assay, *C. longiflorus* stems extract (0.05 mL) or Trolox (as positive control) were added to 1.45 mL of DPPH free radical. The absorbance was measured at 515 nm after 30 min of incubation at room temperature in the dark. The equation given below was used to calculate the inhibition percentage of the DPPH:

(2) $$\mathrm{DPPH Inhibition percentage}=\frac{\mathrm{OD}\left(\mathrm{negative control}\right)-\mathrm{OD}\left(\mathrm{sample}\right)}{\mathrm{OD}\left(\mathrm{negative control}\right)}\times 100$$

The ABTS scavenging capacity was expressed in mM Ascorbic Acid Equivalent. In addition, CUPRAC (cupric ion reducing antioxidant capacity assay kit) and FRAP (ferric reducing antioxidant power assay kit) were used (Bioquochem, Asturias, Spain), to measure the capacity of extracts to reduce cupric ions to cuprous ions in the presence of neocuproine (a chelating agent), and to reduce the ferric ions to ferrous ions in the presence of a complexing agent, respectively [12]. The CUPRAC antioxidant activity was expressed as mM Trolox Equivalent and FRAP results were expressed as μM Iron II equivalent. Each method targets a specific mechanism of antioxidant action, hence all providing a comprehensive evaluation of the antioxidant potential of our samples.

### 2.7. RP-UHPLC-PDA-MS Analysis

Phenolic compounds in the *C*. *longiflorus* IR stem extracts were chromatically separated using a ThermoFisher Vanquish UHPLC system, as described by Hammoud et al. [12]. The flow rate was 0.4 mL/min and the column temperature was 40 °C. Formic acid (0.1% *v*/*v*) in water (A) and in acetonitrile (B) were used as eluents. The following gradient was applied: 0–1 min at 5% B (isocratic), 1–21 min from 5 to 60% B (linear gradient), 21–23 min 60 to 100% B (linear gradient), 23–27 min at 100% B (isocratic), 27–29 min from 100 to 5% B (linear gradient), and 29–34 min at 5% B (isocratic).

Mass spectrometric data was acquired using a LTQ Velos Pro linear ion-trap mass spectrometer (Thermo Scientific, Waltham, MA, USA) equipped with a heated electro-spray ionization (ESI) probe coupled to the UHPLC system. Over the *m*/*z* range of 160–1500, data were collected in both positive and negative ionization modes. Data-dependent MS2 analysis was performed on the most intense ion by using collision-induced dissociation with a normalized collision energy of 35%. To obtain MS2 spectra of multiple ions present in the full MS spectra, dynamic exclusion was used with a repeat count of four, repeat duration of 5 s, and exclusion duration of 5 s. MS settings were optimized by automatic tuning using LTQ tune Plus 4.2 in Xcalibur 4.2 (Thermo Scientific, USA). Nitrogen was used as a sheath gas (50 arbitrary units) and an auxiliary gas (13 arbitrary units). The ion-transfer tube temperature was 263 °C, the source heater 425 °C, and the source voltage was 2.5 kV and 3.5 kV in negative and positive mode, respectively.

### 2.8. Antibacterial Activity

#### 2.8.1. Minimum Inhibitory Concentration (MIC) and Minimum Bactericidal Concentration (MBC) Assays

The microdilution method, as recommended by the CLSI [15], was used to determine MICs and MBCs. Using a 96-well cell culture plate (TPP tissue culture plates, 96 wells, flat bottom, 6.4 MM, Zellkutur und Labortechnologie, Schaffhausen, Switzerland), a 100 μL aliquot of the extract was transferred to complete serial two-fold dilutions in MHB. A diluted bacterial suspension (5 μL) was added to each well in order to achieve a final concentration of 5 × 10^5^ CFU/mL. Positive and negative controls were also performed. Plates were incubated for 24 h at 37 °C. The MIC for every extract corresponds to the lowest concentration, where no visible growth was observed. Finally, in order to determine the MBC for every extract, which corresponds to the lowest concentration by killing >99.9% of the bacterial population; the contents of all wells with no visible growth were plated on BHA.

#### 2.8.2. Antibiofilm Activities

##### Biofilm Formation

The assay of biofilm formation in polystyrene was performed essentially according to a standard procedure [16]. A bacterial strain was grown on TSB supplemented with glucose overnight, and a bacterial inoculum was added to the microplate to reach a concentration of 5 × 10^5^ CFU/mL in all wells, except for column 12, which is used as a negative control. The microplate was incubated for 24 h at 37 °C. Subsequently, the well’s contents were discarded and the biofilms formed were fixed by heating at 80 °C for 1 h. The wells were washed with distilled water to remove non-adherent bacteria. Furthermore, 100 µL of 0.1% (*w*/*v*) crystal violet were added to all wells for 5 min. Optical density (OD) was measured at 570 nm using an ELISA microplate reader (BioTek, Bad Friedrichshall, Germany).

##### Biofilm Eradication Assay

The plates were ready for treatment with the plant extracts after the fixation of the formed biofilm. All wells were filled with 100 μL of sterile distilled water and a serial two-fold dilutions were performed with an equal volume of the extract in the wells, except for column 11, which was used as a positive control. The microplates were incubated for 18 h at 37 °C. Hereafter, the contents of the wells were discarded and filled with 100 μL 0.1% (*w*/*v*) crystal violet for 5 min. Subsequently, the wells were washed with distilled water and the OD was measured at 570 nm using an ELISA microplate reader (BioTek, Germany). The minimal biofilm eradication concentration (MBEC) was defined as the lowest concentration exhibiting the highest significant biofilm eradication, and the percentage of eradication was calculated as follows:

(3) $$\%\;\mathrm{eradication}=\frac{\mathrm{OD}\left(\mathrm{positive control}\right)-\mathrm{OD}\left(\mathrm{treated well}\right)}{\mathrm{OD}\left(\mathrm{positive control}\right)-\mathrm{OD}\left(\mathrm{negative control}\right)}\times 100$$

##### Biofilm Prevention Assay

A 96-well microplate was used for the biofilm prevention activity of *C. longiflorus* stems extracts. A 100 μL aliquot of TSB medium complemented with glucose 0.25% (*w*/*v*) and 100 μL of extract were added to the first well. Then, a serial two-fold dilution was done in TSB till the 10th well. An inoculum of diluted bacterial suspension was added to each well to have a final concentration of 5 × 10^5^ CFU/mL. The remaining steps of fixation, washing, staining, and measuring were carried out as previously described above in the biofilm eradicative activity measurement. The minimal biofilm prevention concentration (MBPC) is described as the lowest concentration having the highest significant prevention, and the percentage of prevention was calculated as follows:

(4) $$\%\;\mathrm{prevention}=\frac{\mathrm{OD}\left(\mathrm{positive control}\right)-\mathrm{OD}\left(\mathrm{treated well}\right)}{\mathrm{OD}\left(\mathrm{positive control}\right)-\mathrm{OD}\left(\mathrm{negative control}\right)}\times 100$$

### 2.9. Statistical Analysis

All measurements and experimentations were done in triplicates, and the results were reported as average values ± standard deviation. All data were analyzed and considered significant when *p*-values < 0.05. The obtained results were processed by ANOVA variance analyses and Least Significance Difference (LSD) using the software STATGRAPHICS^®^ (Centurion XVII-X64). GraphPad Prism^®^ Software (Version 6.05; GraphPad Software, Inc., San Diego, CA, USA) was used for antibiofilm activities analysis.

## 3. Results and Discussion

### 3.1. Optimization of Extraction Parameters and Their Effects on TPC

#### 3.1.1. Effect of Particle Size

**Figure 2:**
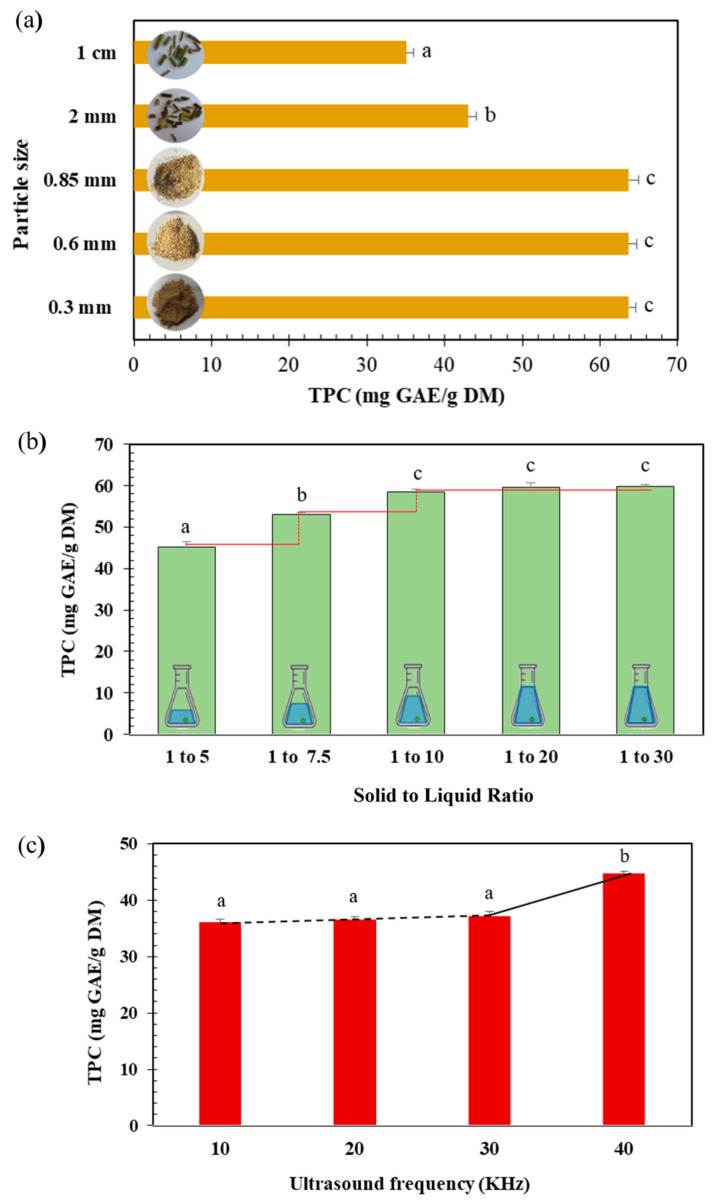
(**a**) Effect of particle size of ground *Centranthus longiflorus* stems on the polyphenol’s extraction yield (TPC mg GAE/g DM) using WB extraction technique. (**b**) Effect of solid to liquid ratio stems on the polyphenols extraction yield (TPC mg GAE/g DM) from *C. longiflorus* stems using WB extraction technique. (**c**) Effect of different ultrasound frequencies on polyphenols extraction yield (TPC mg GAE/g DM) of *C. longiflorus* stems extracts. a, b, and c indicate significant statistical difference (*p* < 0.05).

Decreasing the particle size from 1 cm to 0.85 mm led to an increase in TPC from 35.05 to 63.67 mg GAE/g DM (Figure 2a). A further size reduction did not increase the polyphenol content. Our results are in accordance with previous studies demonstrating that the reduction of particle size strongly influences polyphenols extraction yield by increasing the contact surface and the mass transfer between sample and solvent [17,18]. However, the reduction of particle size to very fine dimensions can lead to clustering and aggregation, thus affecting the extraction process by limiting the overall solvent accessibility [19]. A particle size of 0.85 mm was therefore selected for further experiments.

#### 3.1.2. Effect of Solid to Liquid Ratio

Solid to liquid ratio plays a key role in the optimization of the extraction process. Moreover, excessive solvent volume presents a negative environmental impact [20]. As shown in Figure 2b, the extraction yield increased by 23% while increasing the solid to liquid ratio from 1:5 to 1:10 (g:mL). No significant increase was noticed for higher ratios (1:20 and 1:30). Thus, 1:10 was adopted for all the experiments.

#### 3.1.3. Effect of Ultrasound Frequencies

Increasing US frequencies from 10 to 40 kHz significantly increased the polyphenols content (by 24%) from 36.06 to 44.83 mg GAE/g DM (Figure 2c). This is in agreement with an extraction study demonstrating that an optimum of TPC was obtained using 40 kHz [21]. Hence, a frequency of 40 kHz was chosen for all the subsequent experiments.

### 3.2. Effect of Time, Temperature, and Ethanol Percentage by Response Surface Methodology

On the one hand, RSM was conducted to investigate the effect of the operating conditions in terms of extraction time, extraction temperature and ethanol percentage, on TPC and DPPH values, and on the other hand, to determine their optimal combination for the three extraction techniques (WB, US, and IR). The ultimate goal was to maximize the recovery of TPC from *C. longiflorus* stems and the DPPH inhibition as well. A model was designed after setting the solid to liquid ratio to 1:10 and the particle size to 0.85 mm, while varying time, temperature, and solvent mixture. TPC values and DPPH inhibition percentages for WB, US, and IR extracts are summarized in Table 1.

**Figure 3:**
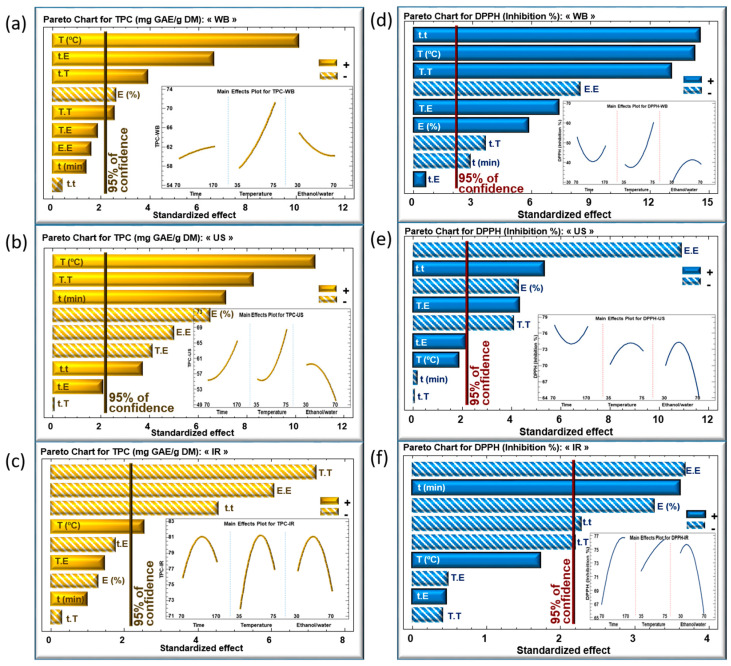
Standardized Pareto charts of TPC and DPPH inhibition percentage using WB (**a**,**d**), US (**b**,**e**) and IR (**c**,**f**) extraction techniques of *Centranthus longiflorus* stems. The variables studied are extraction time “t”, extraction temperature “T”, and solvent mixture “E”. The vertical line indicates statistical significance with more than 95% of confidence. (+) indicates a positive effect, (−) indicates a negative effect.

The impact of the extraction parameters was analyzed according to the Pareto charts for TPC (Figure 3a–c) and DPPH (Figure 3d–f) for WB, US, and IR, respectively. Inserts in these figures show the evolution of the TPC (Figure 3a–c) and DPPH (Figure 3d–f) as a function of the three parameters within the studied domains of variation.

For the extraction time, no effect was detected in case of WB (Figure 3a), whereas its effect was significantly linearly positive in case of US (Figure 3b). Meanwhile, the major effect of extraction time using IR was quadratic negative, which led to a maximum level of TPC within the chosen domain of variation (Figure 3c).

The three extraction techniques showed a significant, positive linear correlation between extraction temperature and TPC, as shown in Figure 3a–c. This finding confirms that increasing the extraction temperature has a pronounced effect on enhancing the phenolic content, resulting in maximal recovery [22]. This linear effect was highly significant when using WB and US, but less significant with IR. A higher extraction temperature boosts the diffusion coefficient and the solubility of phenolic compounds, permitting a greater extraction rate [23]. Many studies reported the efficiency of heat-induced extraction by improving the mass transfer between the sample and the solvent, thus aiding solubilization [24]. Nonetheless, and beyond a certain limit (based on the mode of heating), a very high extraction temperature may degrade and/or oxidize the extracted TPC [23]. This is in line with the quadratic effect of temperature showing a significant, negative impact on TPC when using the IR technique (Figure 3c), which means that the TPC reached a maximum as a function of T.

The ethanol percentage revealed a linear negative effect on TPC in WB and US extraction techniques and no significant effect in IR (Figure 3a–c). Its quadratic effect (E.E) was either negative (US and IR) or not significant (WB). Increasing the ethanol percentage negatively affected the recovery yield of TPC, which is probably related to the solubility of the phenolic compounds in media of different polarities. Previous research discussed the impact of emitted IR wavelength on the efficiency of the solvent depending on its polarity [25,26]. As a consequence, a substantial quadratic negative effect of the solvent was observed, which is reflected by a maximum TPC using 48% of ethanol/water mixture.

The significance level of the parameters with regard to the DPPH is represented in Figure 3d–f for WB, US and IR, respectively. In general, marked effects were not apparent. A positive linear effect of temperature was nevertheless observed in the case of WB, and a negative quadratic effect of the solvent for the three techniques. Regarding this latter observation, a balanced mixture between polar and non-polar solvents would have resulted in the extraction of an assortment of phenolic compounds with high-antioxidative power.

**Figure 4:**
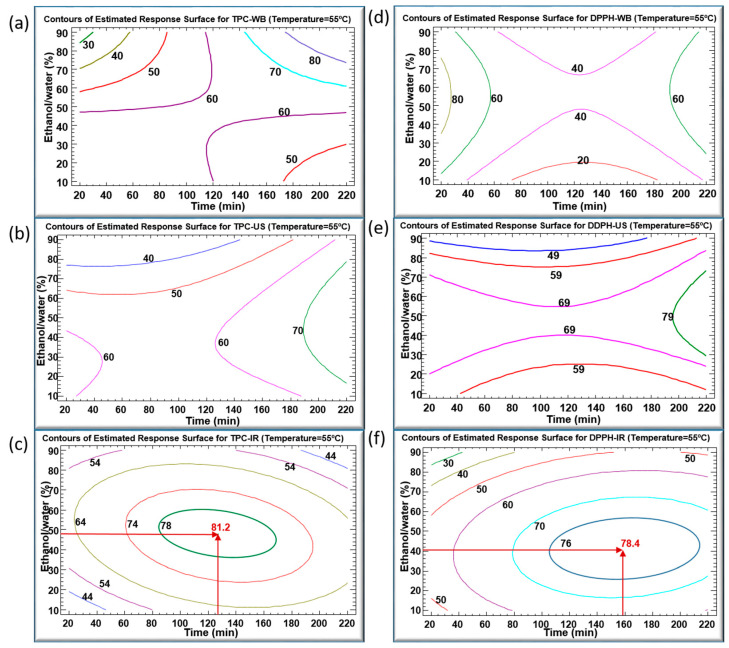
Contours of estimated response surfaces of TPC and DPPH inhibition percentage using WB (**a**,**d**), US (**b**,**e**) and IR (**c**,**f**) extraction techniques of *Centranthus longiflorus* stems. Every colored line indicates the same value of TPC or DPPH.

Figure 4a–f illustrate the contours of the estimated response surfaces for TPC and DPPH of the obtained *C*. *longiflorus* stems extracts using the three extraction techniques. They describe the evolution of TPC and DPPH as a function of ethanol percentage and extraction time, while the temperature was set at 55 °C. These contours indicate a maximum of TPC values (mg GAE/g DM) of 80, 70 and 81.2, and a maximum of DPPH values (%) of 80, 79 and 78.4 for WB, US and IR, respectively. They are composed of an infinite number of possible combinations of ethanol percentage and extraction time, each of which could yield the same value of the variable response. Accordingly, it would be possible to choose the most convenient arrangement: high productivity/low cost, which corresponds to the shortest extraction time, or environmentally friendly/low cost, which requires a minimal use of ethanol as solvent.

Figure 4a reveals a maximal TPC of 80 mg GAE/g DM obtained after an extraction time of 200 min in WB and with an ethanol percentage between 80% and 90%. This maximum coincides with a DPPH inhibition of 60%. A prolonged heating in WB may account for this relatively low antioxidant activity by increasing the amount of the extracted TPC, yet inducing an increased degradation of their quality. Simultaneously, Figure 4d indicates an 80% DPPH obtained with a shorter WB extraction time (20–30 min), but corresponding to TPC values lower than 60 mg GAE/g DM. A lower amount of TPC was extracted during a much shorter time, but demonstrated higher antioxidant activity.

With regard to the sonication in Figure 4b,e, the highest values of TPC (70 mg GAE/g DM) and DPPH (79%) were obtained during a treatment time longer than 200 min and using around 55% ethanol. Here, it is worthy to emphasize the efficiency of US treatment as well. In fact, US improves the extraction yield by generating steam bubbles with expansion–compression cycles, resulting in their collapse which would damage the cell membrane [27]. The penetration of the solvent into the product will rise after cell disruption, thus intensifying the release of the target intracellular compounds. Nevertheless, the downside of high US frequency cannot be neglected, as it may cause degradation of the bioactive compounds [28].

The infrared-assisted extraction was the most efficient method for polyphenols recovery from the *C. longiflorus* stems with a TPC value reaching 81.2 mg GAE/g DM. Our results are in agreement with earlier studies that proved the efficiency of IR as an extraction technique when applied on pomegranate peels [26], olive leaves [29], blood orange peels [30], *Saussurea lappa* roots [31] and *Eryngium creticum* [12], as compared to WB. The high efficiency of the IR extraction might be attributed to the infrared radiation wavelength, which is highly absorbed by the solvent and by the plant’s bioactive compounds [32]. IR irradiation may directly heat the sample-solvent mixture without heating the container [20]. Furthermore, exposure to infrared irradiation can induce both molecular vibrations and structural modifications, which may lead to an enhanced release of polyphenols from plant materials [32].

The maximal TPC obtained using IR (81.2 mg GAE/g DM) was reached using 48% ethanol at 55 °C for 127 min. At such mild conditions, this TPC was higher than using WB and US, showing corresponding TPC values of about 63 and 62 mg GAE/g DM, respectively. In addition, the IR extraction technique resulted in the highest DPPH inhibition percentage of 77%, compared to 41% and 74% obtained by WB and US, respectively. The maximums recorded in the case of IR can be considered as optimums standing at the center of the domain of variations of parameters. Every multiple optimum resulted from the combination of two separate optima: the ethanol percentage and extraction time (which both had negative quadratic effects).

**Figure 5:**
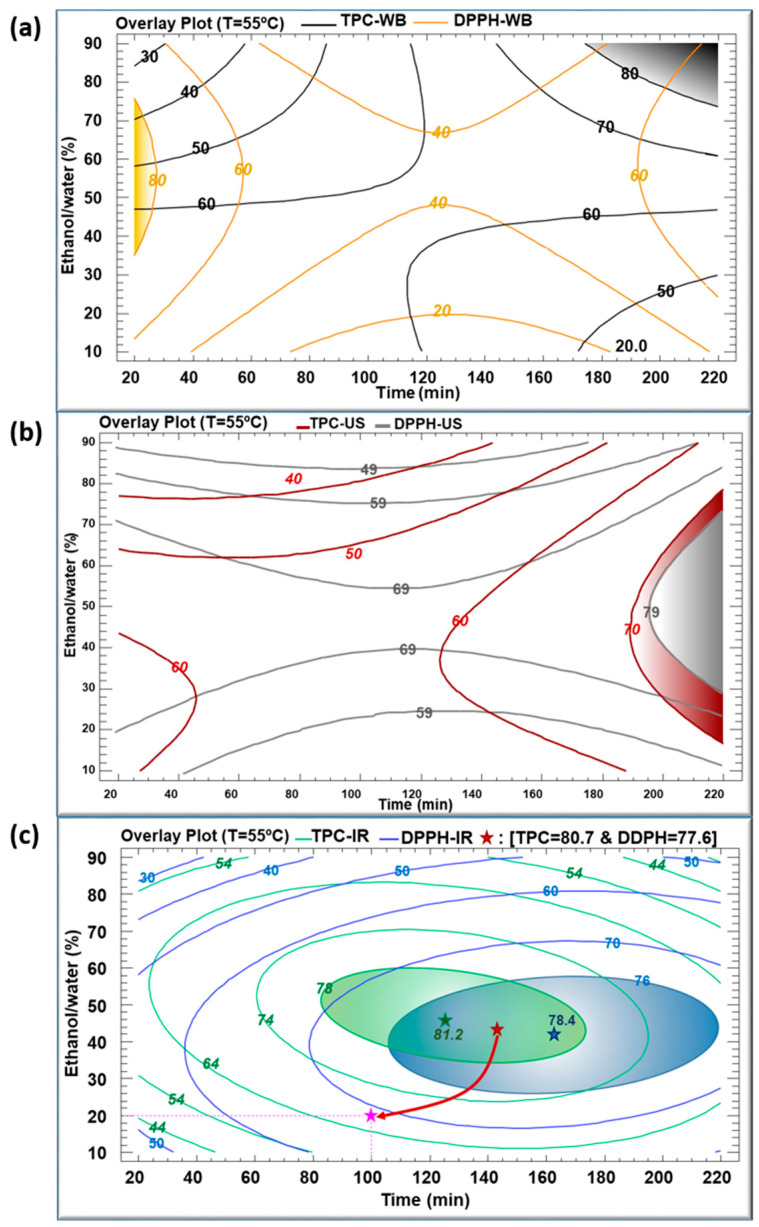
The overlay plots of the multiple response of all parameters using WB (**a**), US (**b**) and IR (**c**) extraction techniques of *Centranthus longiflorus* stems. The colored lines indicate the value of TPC and DPPH inhibition. Green star, blue star and red star represent the optimum of TPC, the optimum of DPPH, and the multiple optimum, respectively, for the IR extraction method.

Figure 5a–c illustrate the multiple response optimizations for TPC and DPPH using the three extraction techniques with a temperature set at 55 °C. In Figure 5a, related to WB, it is noticeable that the gray region (upper right corner) corresponds to an extraction time of more than 200 min with an ethanol percentage above 90%, that resulted in the maximum TPC. Meanwhile, the maximum DPPH inhibition percentage required 20 min to preserve the antioxidant activity of phenolic compounds, using 50% ethanol–water mixture (yellow region). Since the optimum regions are set clearly apart, a multiple optimization would be a compromise between the polyphenols concentration and their quality in terms of DPPH. A maximum TPC of 80 mg GAE/g DM can be reached with a relatively low antioxidant activity, whereas a high DPPH (90%) is reached with a TPC varying between 40 and 60 mg GAE/g DM.

Figure 5b shows the optimum regions of TPC of 70 mg GAE/g DM and 79% DPPH colored in red and gray, respectively. Using the US extraction technique, both optimum conditions overlap. These optima of TPC and DPPH can be reached using 55% ethanol percentage for an extraction time longer than 200 min, which corresponded to a relatively low yield with a high cost.

The combination of the quadratic negative effects of t and E, as shown in inserts of Figure 3c,f, resulted in TPC and DPPH optimums at the center of the variation domain of the operating parameters, as shown in Figure 4c,f. In Figure 5c, the green oval represents the optimum region for TPC (green star corresponding to 81.2 mg GAE/g DM), and the blue oval represents the optimum region of DPPH (blue star corresponding to 78.4%). Both TPC and DPPH optimums overlapped, exhibiting a common area. As a balanced multiple optimum, the red star (145 min, 42% ethanol) represents a TPC of 80.7 mg GAE/g DM and a DPPH of 77.6%. Moreover, the response–surface method permits some leeway in the choice of the operating parameters, depending on the requirements in terms of quality and cost. For instance, by selecting a response point (pink star) corresponding to a TPC of 70 mg GAE /g DM and a DPPH of 70%, the heating time can be reduced from 140 to 100 min and the ethanol percentage from 42 to 20%. This not only results in substantial energy savings (40% less) but also in solvent consumption reduction (50% less).

In conclusion, extraction by IR resulted in a better recovery of TPC with higher antioxidant activity as compared to WB. On another note, although the optimum obtained by IR only slightly exceeded the one obtained by US, this latter required a much longer time than IR (38% more).

Table 2 shows the equations generated by the experimental design analysis to predict TPC and DPPH values of extracts obtained using the three techniques.

The statistical analyses confirm the fitting of measured response values with the suggested second order polynomial models with a high coefficient of determination R^2^, ranging between 90% and 98%.

### 3.3. Antiradical and Antioxidant Activities of C. longiflorus Extracts

**Figure 6:**
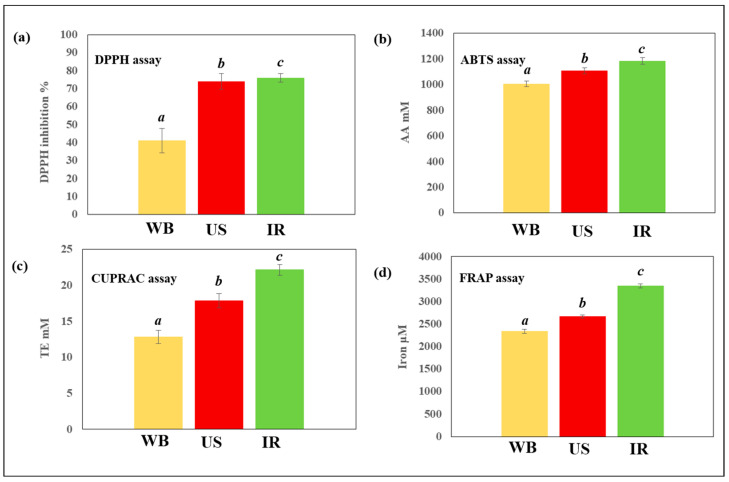
Antiradical and antioxidant activities of *Centranthus longiflorus* stem extracts obtained using different extraction techniques (WB, US and IR), and assessed by (**a**) DPPH, (**b**) ABTS, (**c**) CUPRAC, and (**d**) FRAP assays. a, b, and c indicate significant statistical difference between means.

The antiradical and antioxidant capacities of *C. longiflorus* stem extracts which were obtained under the same conditions using the three extraction techniques, were assessed and compared (Figure 6). *C. longiflorus* IR extract showed the highest antiradical capacity, followed by US and WB (76%, 74% and 41% DPPH inhibition, respectively). Likewise, the IR extract revealed the highest antioxidant capacity, as studied by ABTS, CUPRAC, and FRAP assays. This observation may possibly be explained by the high-phenolic content obtained by the IR (81 mg GAE/g DM), compared to other studied extraction techniques. However, and since a high TPC in a plant extract does not necessarily correspond to a high-DPPH value (see Table 1), the high-antioxidant capacity observed could depend on the specific combination or collection of polyphenols extracted.

### 3.4. Antibacterial Activity of C. longiflorus Extracts

All *C. longiflorus* stem extracts demonstrated the same overall low antibacterial effect against the tested strains regardless of their types. The MIC and MBC recorded were 50 mg/mL and 100 mg/mL, respectively (Table 3).

Numerous studies have focused on the antibacterial capacity of the genus *Centranthus* extracts. Makki et al. [33] investigated *C. longiflorus* extracts obtained after maceration, with water or ethanol, for 8 h at room temperature, regarding their antibacterial capability. The aqueous extract exhibited an antibacterial activity against *S. epidermidis* CIP 444 (MIC equal to 428 mg/mL) with no detectable MBC at the maximal concentration used. The same value, 450 mg/mL, was registered for MIC and MBC against *P. aeruginosa*. However, no detectable MIC and MBC against *S. aureus*, *E. coli* and *E. faecalis* for the aqueous extract was found. The ethanolic *C. longiflorus* extract proved the most effective against *S. epidermidis* CIP 444. The MIC registered was 160 mg/mL, but no detectable MBC was found. The same effect was shown against *P. aeruginosa*, where MIC = MBC = 400 mg/mL. In addition, a similar effect was obtained against *S. aureus* and *E. coli* (450 mg/mL) with no detectable MBC at the maximal concentration used.

A previous study demonstrated a stronger antibacterial activity of *C. longiflorus* extract, grown in Turkey and extracted by Soxhlet. The obtained MIC was equal to 3 mg/mL against *P. aeruginosa* [34]. In contrast, no detectable MICs of *C. longiflorus* against *P. aeruginosa*, *S. aureus* and *E. coli* was observed using the ultrasound extraction technique [35]. Such discrepancies in the antibacterial capacities of plant extracts can be attributed to multiple factors, such as the geographical location, the season of collection of samples, the bacterial strains used, and the various bioactive compounds of the extract.

### 3.5. Antibiofilm Activity of C. longiflorus Extracts

Bacteria can exist in several modes during their life cycle; they can exist in a planktonic (free-living) state or in a structured sessile form known as a biofilm. Bacterial biofilm is a community of microorganisms that are attached to a surface and embedded in their matrix [36,37]. Their ability to form biofilm will make them more resistant to antibiotic treatment and host defense mechanisms, which complicates the process of their elimination [38]. Therefore, it is of utmost importance to seek new antibiofilm agents to eradicate already formed biofilms or prevent their formation.

#### 3.5.1. Biofilm Eradication Activity

**Figure 7:**
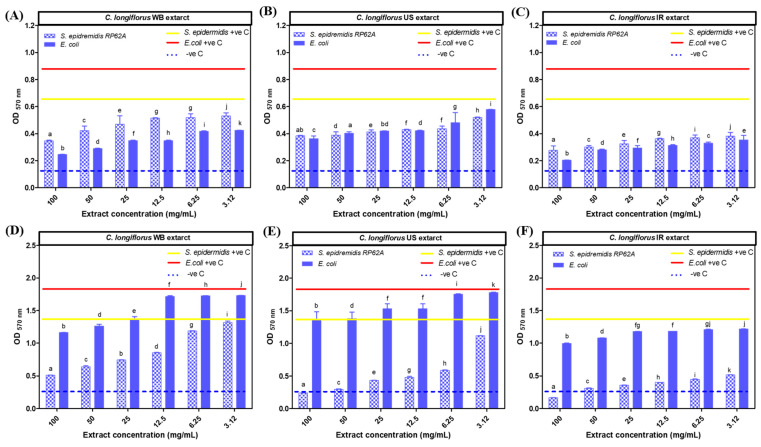
Results of the different extracts showing crystal violet optical density (OD 570 nm) in function of extracts concentrations. (**A**–**C**): antibiofilm eradication activity of *Centranthus longiflorus* stem extracts on *Staphylococcus epidermidis* RP62A and *Escherichia coli* strains. (**D**–**F**): antibiofilm prevention activity of *C*. *longiflorus* stem extracts on *S*. *epidermidis* RP62A and *E*. *coli* strains. There was a significant difference for the treated wells compared to the untreated ones. −ve C is crystal violet optical density of blank. a–k indicate significant statistical difference (*p* < 0.001).

The results shown in Figure 7 confirmed that all *C. longiflorus* stem extracts (WB, US and IR) exhibited an eradication potential with a higher antibiofilm capacity against *E. coli*, compared to *S. epidermidis* biofilms, as shown in Table 3. In addition, *C. longiflorus* IR extract exhibited the highest biofilm eradication capacity against *E. coli* biofilm. The IR extract was able to eradicate 93% *E. coli* biofilms, compared to 87% for the WB extract and 73% for the US extract at the same concentration (Figure 7A–C).

#### 3.5.2. Biofilm Prevention Activity

In contrast to the biofilm eradication activity, *C. longiflorus* stem extracts had a more preventive effect against *S. epidermidis* biofilms than against those of *E. coli*. *C. longiflorus* IR extract exhibited the strongest prevention capacity against *S. epidermidis* biofilm with a preventive percentage of about 97%, followed by the US extract (94%) and the WB extract (80%) as shown in Figure 7E,F. All these prevention percentages were reported at the same highest extract concentration used (100 mg/mL). In conclusion, *C. longiflorus* extracts seem to be more preventive against *S. epidermidis* and more eradicative against *E. coli*.

### 3.6. Identification of Phenolic Compounds by RP-UHPLC-PDA-MS

**Figure 8:**
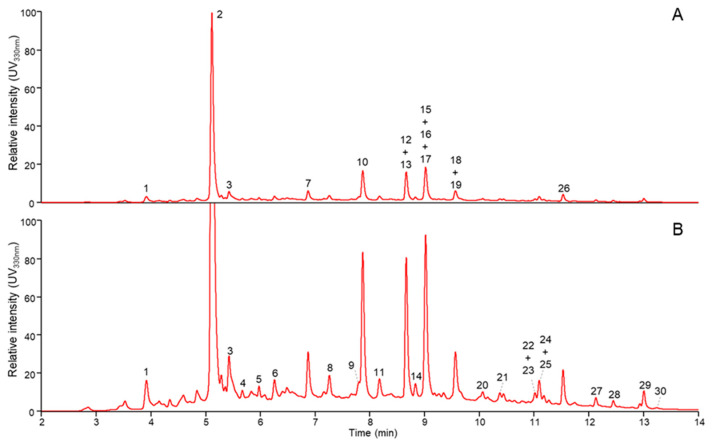
RP-UHPLC-UV elution profiles (330 nm) at full (**A**) and 5× zoomed intensity (**B**). Numbers refer to tentatively identified compounds shown in Table 4.

Phenolic compounds in the IR extract, obtained under the experimental conditions corresponding to the central points (Table 1, runs 15 to 22), were identified by RP-UHPLC-PDA-MS (Figure 8). The analysis was based on retention time, λ max and fragmentation and compared to the literature [39,40,41].

Many different flavonoids were present in the extract with caffeic acid derivatives, flavone and flavonol glucosides and their aglycones as predominant compounds (Table 4).

The chromatographic screening gradient that was used allowed the direct mapping of diverse phenolic compounds. Due to this screening gradient, several related and structurally close compounds co-eluted. Although, helped by the dependent scan fragmentation operation of the MS, the analysis did allow the tentative identification of most compounds present (Table 4). Based on UV intensity, 3-*O*-caffeoylquinic acid (2) and quercetin rutinoside (10) were the most abundant compounds in the extract.

Numerous studies in the literature correlate the biological properties of medicinal plants to their phytochemical profile. Caffeoylquinic acid and its derivatives are well known as potential antioxidants [42], in addition to their significant antimicrobial, antitumor, anti-inflammatory activities [43]. 3-*O*-caffeoylquinic acid, the major active compound identified in *C. longiflorus* stems IR extract, exhibits high-antioxidant activity. These significant biological properties of the family of caffeoylquinic acid may lead to several possible applications of the plant extract, as a food preservative of natural origin, or as a potential resource for natural drugs. Furthermore, quercetin and related compounds are known for their health benefits displaying a widespread range of biological activities, including powerful anti-inflammatory, anticancer, antioxidant, antibacterial, and antiviral capabilities, leading to pharmaceutical, cosmetic, and food industry applications [44].

## 4. Conclusions

The goal of this study was to investigate the chemical composition and the biological activities of *C*. *longiflorus* stem extracts recovered using water bath, ultrasound and infrared extractions. The effectiveness of *Ired-Irrad*^®^ as a green alternative method for polyphenols’ extraction was demonstrated. The final phenolic concentration was increased using the infrared-assisted extraction in comparison to the conventional water bath and ultrasound treatment. The bioactivities of the extract, i.e., antioxidant and antibacterial capacities, were improved as well. The strongest biofilm eradication and prevention capacities (against *E. coli* and *S. epidermidis*, respectively), were exhibited by the IR extract. The RP-UHPLC-PDA-MS analysis revealed that the most abundant compounds in the *C. longiflorus* stem’s IR extract were 3-*O*-caffeoylquinic acid and quercetin rutinoside, which were the key contributors to the extract’s bioactivity. Upon optimization, *Ired-Irrad*^®^ required a minimal consumption of organic solvent for a relatively shorter extraction time. It was validated as a simple, cost-efficient and environmentally friendly technique that can be applied for the extraction of plant bioactive compounds.

## 5. Patent

Rajha, H.N., Debs, E., Maroun, R.G., Louka, N. “System for extracting, separating or treating products through infrared radiation. Adequacy between the properties of infrared radiation and those of the processed products”. Lebanese patent number 2017/11-11296L granted on 29 November 2017.

## Figures and Tables

**Table 1:** Central composite design of independent parameters and their response variables TPC (mg GAE/g DM) and DPPH inhibition percentage for *Centranthus longiflorus* WB, US and IR stem extracts.

	Run	Independent Parameters			Response Variables					
		Time (min)	Temperature (°C)	Ethanol Percentage (%)	WB		US		IR	
					TPC (mg GAE/g DM)	DPPH Inhibition %	TPC (mg GAE/g DM)	DPPH Inhibition %	TPC (mg GAE/g DM)	DPPH Inhibition %
Factorial design	1	70	35	30	71.50	44.54	50.75	71.73	61.10	56.62
	2	170	35	30	52.96	45.64	59.25	73.28	65.57	71.12
	3	70	75	30	80.26	54.47	73.49	71.59	57.56	79.48
	4	170	75	30	73.53	47.04	77.83	62.8	67.34	74.38
	5	70	35	70	45.30	37.34	48.8	60.13	60.35	51.73
	6	170	35	70	51.50	46.63	61.15	59.21	62.69	65.46
	7	70	75	70	56.02	81.98	52.3	65.19	71.24	67.16
	8	170	75	70	87.96	71.12	68.23	74.12	65.08	69.28
Star points	9	36	55	50	58.01	76.52	57.96	85.23	69.25	53.50
	10	204	55	50	60.75	63.72	74.99	83.75	71.06	78.27
	11	120	21	50	51.81	47.77	59.20	65.15	56.50	76.57
	12	120	88	50	81.46	86.69	91.06	69.76	69.95	68.65
	13	120	55	16	62.87	20.07	59.60	60.87	73.67	70.49
	14	120	55	83	65.75	28.59	40.26	49.41	58.71	51.17
Central points	15	120	55	50	60.93	41.11	58.94	74.04	82.21	74.45
	16	120	55	50	62.08	39.18	59.33	73.91	80.62	74.09
	17	120	55	50	60.62	41.34	57.92	74.70	81.19	74.80
	18	120	55	50	60.88	40.19	55.53	75.43	80.61	74.52
	19	120	55	50	61.10	40.98	58.09	74.97	80.66	74.17
	20	120	55	50	61.77	39.64	56.63	73.78	80.97	75.58
	21	120	55	50	61.50	41.25	58.49	70.69	81.19	74.66
	22	120	55	50	60.66	40.69	57.61	74.51	80.35	75.02

**Table 2:** Second order polynomial equations for TPC and DPPH inhibition percentage corresponding to each extraction technique with the R-squared of each equation.

Extraction Technique	R^2^ (%)	Equation
WB	94	TPC = 156 − 0.59·t − 0.98·T − 1.72·E − 0.00015·t^2^ + 0.0047·t·T + 0.008·t·E + 0.0055·T^2^ + 0.0056·T·E + 0.0034·E^2^
	98	DPPH = 126 − 0.86·t − 2.47·T + 0.64·E + 0.004·t^2^ − 0.0036·t·T + 0.0006·t·E + 0.023·T^2^ + 0.018·T·E − 0.015·E^2^
US	97	TPC = 56 − 0.23·t − 0.65·T + 0.9·E + 0.001·t^2^ − 0.00007·t·T + 0.002·t·E + 0.014·T^2^ − 0.0096·T·E − 0.00839598·E^2^
	95	DPPH = 71.7 − 0.42·t + 0.31·T + 0.84·E + 0.0014·t^2^ − 0.00006·t·T + 0.0019·t·E − 0.0066·T^2^ + 0.0096·T·E − 0.017·E^2^
IR	91	TPC = −37.7 + 0.56·t + 1.77·T + 1.35·E − 0.0017·t^2^ − 0.0004·t·T − 0.0023·t·E − 0.017·T^2^ + 0.0047·T·E − 0.014·E^2^
	90	DPPH = −9.3 + 0.55·t + 0.84·T + 0.97·E − 0.0012·t^2^ − 0.0039·t·T + 0.0008·t·E − 0.013·T^2^ − 0.002·T·E − 0.012·E^2^

**Table 3:** Antibacterial and antibiofilm eradication and prevention activities (as percentage) of *Centranthus longiflorus* WB, US and IR stem extracts. The bacterial strains marked with an asterisk were only tested for their antibacterial activity.

		Antibacterial Activity		Antibiofilm Activity											
				Eradication (%)						Prevention (%)					
Technique	Bacterial Strains	MIC mg/mL	MBC mg/mL	100	50	25	12.5	6.25	3.12	100	50	25	12.5	6.25	3.12
WB	*S. epidermidis*	50	100	72	63	57	52	51	50	80	73	68	63	46	39
	*E. coli*	50	100	87	82	74	76	67	66	69	64	59	41	35	26
	P. aeruginosa *	50	100												
	S. aureus *	50	100												
US	*S. epidermidis*	50	100	68	67	64	62	61	51	94	91	84	82	76	49
	*E. coli*	50	100	73	68	67	66	59	47	59	58	50	50	35	30
	P. aeruginosa *	50	100												
	S. aureus *	50	100												
IR	*S. epidermidis*	50	100	80	78	75	70	69	68	97	90	88	86	83	80
	*E. coli*	50	100	93	83	81	79	77	74	77	73	68	68	67	66
	P. aeruginosa *	50	100												
	S. aureus *	50	100												

**Table 4:** Tentatively identified phenolic compounds in *Centranthus longiflorus* IR stem extract.

No.	R_t_ UV (min)	λ_max_ (nm)	R_t_ MS (min)	[M-H]^−^ (*m*/*z*)	MS^2^ Fragments ^a^	Tentative Annotation ^b^
1	3.93	322	4.02	353	191, 179, 135	1-*O*-caffeyolquinic acid
2	5.12	326	5.21	353	191, 179, 135	3-*O*-caffeyolquinic acid
3	5.43	326	5.52	353	191, 179, 135	4-*O*-caffeyolquinic acid
4	5.68	322	5.77	179	135	Caffeic acid
5	5.98	346	6.09	755	593, 285, 447	Luteolin glycoside
6	6.28	318	6.37	337	191, 163	*p*-Coumaroylquinic acid
7	6.88	326	6.97	367	191, 173, 193	5-Feruloylquinic acid
8	7.26	354	7.37	741	300, 609, 591	Quercetin triglycoside
9	7.81	330	7.90	359	197, 153, 135	?
10	7.87	354	7.97	609	301, 343, 271	Quercetin rutinoside
11	8.18	354	8.26	463	301, 343, 179	Quercetin glucoside
12	8.67	346	8.74	507	461, 179, 377	?
13	8.67	346	8.75	593	285, 327, 257	Luteolin glycoside
14	8.84	330	8.94	623	315, 300, 577	Isorhamnetin glycoside
15	9.02	330	9.10	447	284, 285, 327, 255	Luteolin glucoside
16	9.02	n.d.	9.11	515	353, 447, 191	Dicaffeoyl quinic acid
17	9.02	n.d.	9.12	505	459, 265, 193	?
18	9.57	326	9.66	515	353, 299, 202	Dicaffeoyl quinic acid
19	9.57	n.d.	9.68	359	193, 295, 211	?
20	10.06	334	10.17	693	651, 301, 609	Quercetin diacetyl diglycoside
21	10.38	334	10.47	693	651, 301, 609	Quercetin diacetyl diglycoside
22	11.02	330	11.14	359	179, 161, 135, 315	Rosmarinic acid isomer
23	11.02	n.d.	11.14	677	635, 285	Luteolin diacetyl diglycoside
24	11.10	330	11.12	359	179, 161, 135, 315	Rosmarinic acid isomer
25	11.10	n.d.	11.12	677	635, 285	Luteolin diacetyl diglycoside
26	11.53	334	11.62	637	591, 283	Acacetin glycoside
27	12.13	354	12.23	735	693, 651, 301, 463	Quercetin triacetyl diglycoside
28	12.45	346	12.55	851	809, 719, 579, 284	Luteolin triacetyl triglycoside
29	13.01	350	13.09	719	677, 285, 635	Luteolin triacetyl diglycoside
30	13.24	334	13.34	285	285, 151, 257	Luteolin

^a^: fragments in decreasing intensity order; ^b^: based on fragmentation, retention time, λ_max_ compared to literature [38,39,40], n.d.: λ-max could not be determined, ?: compound could not be identified based on MS fragmentation pattern, retention time and λ-max.

## Data Availability

Data available on request.
